# *In vitro* inhibition of Cyprinid herpesvirus-3 replication by RNAi

**DOI:** 10.1016/j.jviromet.2014.05.022

**Published:** 2014-09-15

**Authors:** Michael Gotesman, Hatem Soliman, Robert Besch, Mansour El-Matbouli

**Affiliations:** a Clinical Division of Fish Medicine, University of Veterinary Medicine, Vienna, Austria; b Fish Medicine and Management, Faculty of Veterinary Medicine, University of Assiut, 71515 Assiut, Egypt; c Clinic and Policlinic for Dermatology and Allergology, Department of Dermatology, Ludwig-Maximilian University, Munich, Germany

**Keywords:** CyHV-3, KHV, RNAi, Inhibition

## Abstract

Cyprinid herpesvirus-3 (CyHV-3) is an etiological agent of a notifiable disease that causes high mortality rates affecting both the common and koi carp *Cyprinus carpio* L. There is no current treatment strategy to save CyHV-3 infected fish. RNA mediated interference (RNAi) is an emerging strategy used for understanding gene function and is a promising method in developing novel therapeutics and antiviral medications. For this study, the possibility of activating the RNAi pathway by the use of small interfering (si)RNAs was tested to inhibit *in vitro* viral replication of CyHV-3 in common carp brain (CCB) cells. The siRNAs were designed to target either thymidine kinase (TK) or DNA polymerase (DP) genes, which both code for transcripts involved in DNA replication. The inhibition of viral replication caused by the siRNAs was measured by a reporter gene, termed ORF81. Treatment with siRNA targeting either TK or DP genes reduced the release of viral particles from infected CCB cells. However, siRNA targeting DP was most effective at reducing viral release as measured by qPCR.

## Highlights

• Cyprinid herpesvirus-3 causes high mortality rates in common and koi carp.
• siRNAs were designed to target thymidine kinase and DNA polymerase genes *in vitro*.
• siRNA targeting DNA polymerase gene was most effective at reducing viral release.
• The inhibition of viral replication by the siRNAs was quantitated by qPCR.

Cyprinid herpesvirus-3 (CyHV-3) is an etiological agent of a notifiable disease that causes high mortality rates (80–100%) affecting both the common and koi carp *Cyprinus carpio* L. (Hedrick et al., 2000, Ilouze et al., 2006). Outbreaks of this virus were reported in Europe (Bretzinger et al., 1999), US and Israel (Hedrick et al., 2000), as well as in South East Asia (Sano et al., 2004, Kurita et al., 2009, Han et al., 2013). Two different prophylactic strategies have been developed for protection against CyHV-3: (1) a brief exposure to the virus, followed by transferring infected fish to non-permissive temperature (30 °C) or (2) infection with a live attenuated CyHV-3 (Ronen et al., 2003, Perelberg et al., 2005). However, recent studies indicate that survivors of CyHV-3 infection can become carriers for the disease (St-Hilaire et al., 2005, Bergmann and Kempter, 2011, Eide et al., 2011). There is no current treatment for CyHV-3 infected fish.

RNA mediated interference (RNAi) is an emerging strategy used for understanding gene function and is a promising method in developing novel therapeutics and antiviral medications (Gavrilov and Saltzman, 2012). RNAi based therapeutics have been suggested for the development of novel therapies against viral diseases and parasitic agents of aquatic organisms (Lima et al., 2013). Recent *in vitro* (Sarathi et al., 2008) and *in vivo* (Sarathi et al., 2010) studies have demonstrated promising results for using RNAi to combat white spot syndrome virus (WSSV), which is an aquatic viral disease of shrimp. Similarly, several *in vitro* (Ruiz et al., 2009, Kim and Kim, 2011, Kim et al., 2012) and *in vivo* (Schyth et al., 2007, Schyth et al., 2012, Bohle et al., 2011) studies have tested controlling a fish viral disease, termed viral hemorrhagic septicemia virus (VHSV), by RNAi. For this study, the feasibility of using short double stranded RNAs termed small interfering (si)RNAs was tested to inhibit *in vitro* viral replication of CyHV-3. The siRNAs target either thymidine kinase (TK) or DNA polymerase (DP) genes, which both code for transcripts involved in DNA replication and are regulated differentially when CyHV-3 infected common carp brain (CCB) cells are subjected to non-permissive temperature (Dishon et al., 2007).

**Fig. 1:**
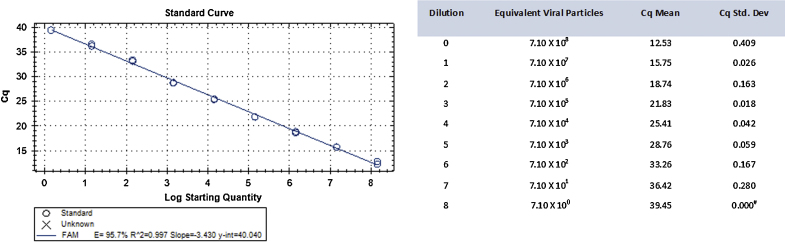
Standard curve for quantitation of ORF81. (A) TaqMan hydrolysis qPCR primer set targeting ORF81 was established for quantitation of the CyHV-3. (B) The dilution series begins with the equivalent of 7.10 × 10^8^ viral particles which is detected below the 13th curve threshold (Cq) and ends with the equivalent of 7.10 × 10^0^ viral particles which is detected near the 40th Cq. The final dilution lacks a duplicate, indicated by #.

A new primer set was designed to measure the release of CyHV-3 from infected cells based on a component of the viral envelope (glycoprotein ORF81), one of the earliest characterized CyHV-3 proteins (Rosenkranz et al., 2008). The 771 bp coding sequence of CyHV-3 ORF81, GenBank access number JQ308818.1, Gene ID: 382929299 was cloned into pET100 (Life Technologies, Wien, Austria). The vector was digested with ScaI (Promega, Wien, Austria) and subjected to a series of 10-fold dilutions starting with 1 ng/μl of plasmid DNA. A standard curve for a primer set targeting ORF81 (Table 1A) was tested for detection of CyHV-3 by TaqMan hydrolysis quantitative (qPCR) and showed the primers to be efficient for quantitation of the virus (Fig. 1). The siRNAs to target CyHV-3-U thymidine kinase gene, GenBank access number AB375385.1, Gene ID: 241661587 and DNA polymerase gene, GenBank access number AY939862.1, Gene ID: 61696088 were designed using Block-iT RNAi Designer (Invitrogen, Wien, Austria), and synthesized by Ambion (Invitrogen) to carry dTdT 3′ overhangs (Table 1B). In addition, siRNAs targeting non-CyHV-3 genes, but a gene in spring viremia of carp virus (SVCV) was used as a control (Table 1C). Duplexes were resuspended in DEPC-treated water to obtain 20 μM (0.266 μg/μl) solutions and aliquoted for use.

**Table 1:** Sequence list (A): primer and probe set targeting the CyHV-3 ORF81 gene (GenBank access number JQ308818, Gene ID: 382929299) used to quantitate CyHV-3 by qPCR. (B): CyHV-3 specific siRNAs target CyHV-3 thymidine kinase (TK) gene (GenBank access number AB375385, Gene ID: 241661587) and DNA polymerase (DP) gene (GenBank access number AY939862, Gene ID: 61696088). (C): Control siRNAs target Spring Viremia of Carp (SVC) virus, nucleoprotein (N) gene (GenBank access number NC_002803, Gene ID: 921324).

Name	Targeted gene	Description	Sequence
A			
ORF81-FP	ORF81	Forward primer	TGCTGTGTTGCTTGCACTTATYT
ORF81-RP	ORF81	Reverse primer	TCAAACKAARGACCGCATTTCG
ORF81-PR	ORF81	Probe	FAM-ATGAAGARGAGTAAACKGCCTGCAACAGA-BHQ1
B			
DP	DNA polymerase	Forward siRNA	CCUCUACAACGUGCACUUUTT
DP	DNA polymerase	Compliment siRNA	AAAGUGCACGUUGUAGAGGTT
DP	DNA polymerase	Gene target	CCTCTACAACGTGCACTTT
TK	Thymidine kinase	Forward siRNA	UCGACGAGGGACAGUUCUUTT
TK	Thymidine kinase	Compliment siRNA	AAGAACUGUCCCUCGUCGATT
C			
TK	Thymidine kinase	Gene target	TCGACGAGGGACAGTTCTT
SVCV-N	Nucleoprotein	Forward siRNA	GGGAUAGCUUCGGACACAATT
SVCV-N	Nucleoprotein	Compliment siRNA	UUGUGUCCGAAGCUAUCCCTT
SVCV-N	Nucleoprotein	Gene target	GGGATAGCTTCGGACACAA

**Fig. 2:**
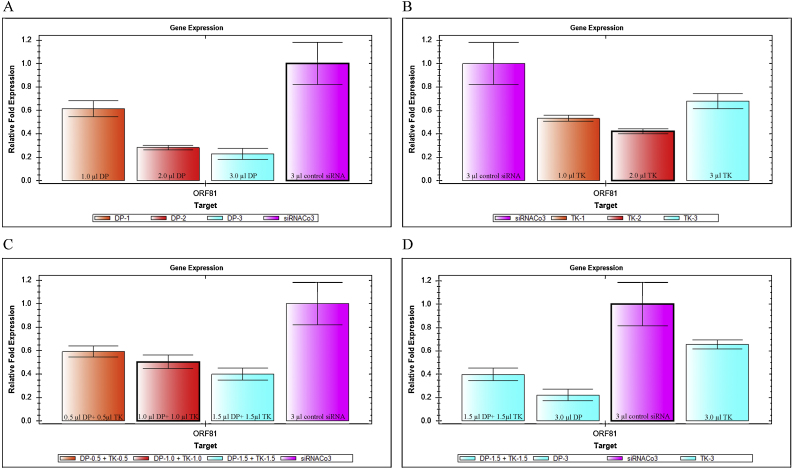
Inhibition of viral replication. For figures (A–D), Taqman qPCR was used to measure the inhibition of viral replication. The *y*-axis refers to relative gene copy number of ORF81 as compared to the sample with 3 μl control siRNA treatment (siRNACo3), the *x*-axis refers to the volume and type of siRNA administered, DNA polymerase (DP) & thymidine kinase (TK) as described in the text. The volume 1 μl is equivalent to 20 μM (0.266 μg/μl), 2 μl is equivalent to 40 μM (0.532 μg/μl), and 3 μl is equivalent to 60 μM (0.798 μg/μl) of siRNA duplexes.

CCB-816 cells were propagated in ZB4G medium: MEM Earle's salts 880 ml/L (Invitrogen), l-Glutamine 200 mM (Invitrogen) 10 ml/L, Gibco non-essential amino acids 10 ml/L (Invitrogen), with 10% per volume fetal bovine serum (FBS) and antibiotic/antimycotic mix (Sigma–Aldrich, Vienna, Austria). Two days after seeding CCB cells into fresh 24-well plates, a 100 μl aliquot of CyHV-3 at 10^3.39^ TCID_50_/ml was added to each plate and incubated at 22 °C. The next day, siRNA aliquots were added according to the manufacturer's instructions into duplicate wells in the previously prepared 24-well plate. Briefly, siRNA aliquots were resuspended in 100 μl opti-mem^®^ I reduced-serum medium liquid (Invitrogen) and incubated with 1 μl Lipofectaimine^®^ LTX reagent (Invitrogen) at room temperature for 30 min and appropriate aliquots were applied immediately into individual wells. On the next day, 24 h post addition of siRNAs, the medium was replaced. Six days later, the medium from each plate was collected to be used for quantitation of viral release by qPCR. DNA was extracted from 200 μl supernatant fractions using the DNeasy Blood and Tissue kit (Qiagen, Hilden, Germany). DNA expression was measured in duplicates using qPCR using the CFX real-time system attached to a C100 Touch thermal cycler (Bio-Rad, Vienna, Austria). For treatment with siRNAs targeting CyHV-3, except for treatment with 3 μl TK, there was a correlative effect for the quantity of siRNA treatment targeting either TK or DP and reduction of viral release as measured by qPCR compared to control siRNAs (Fig. 2). Treatment with a final concentration of 60 nM (3 μl) of siRNAs targeting DP was more effective at reducing virus release than the equivalent treatment targeting TK (3 μl), or a cumulative final concentration for both siRNAS of 60 nM provided by treatment with 1.5 μl DP and 1.5 μl TK (Fig. 2d).

The 295 kb genome of CyHV-3 codes for 156 open reading frames (Aoki et al., 2007) and the relative transcriptional timing for each gene has been annotated (Ilouze et al., 2012). Michel et al. (2010) have elucidated 40 of the CyHV-3 proteins incorporated into mature virons. Several recent studies addressed the host–pathogen interactions of CyHV-3 on the protein level (Gotesman et al., 2013a, Gotesman et al., 2013b, Ouyang et al., 2013) or on the transcriptional level (Adamek et al., 2012, Adamek et al., 2013, Rakus et al., 2012). Infectious CyHV-3 material remains persistent in the natural environmental (Minamoto et al., 2011) and factors such as feeding (Kielpinski et al., 2010), mating (Uchii et al., 2011) or high stocking density (Dishon et al., 2005) exacerbate conditions for CyHV-3 outbreaks. The only currently acceptable measure for the control of CyHV-3 outbreaks is depopulation (eradication of the infected and exposed fish) and disinfection of all materials and systems that have been in contact with infected fish. The purpose of this study was to test the feasibility of using RNAi as an alternative strategy to save CyHV-3 infected fish. The machinery for RNAi is presumed to have developed naturally as a defensive mechanism against viruses and transposable elements (Obbard et al., 2009). Fire et al. (1998) first demonstrated that the RNAi pathway is induced by double stranded RNA. In this study, short double stranded RNA that targets CyHV-3 genes involved in DNA synthesis was tested to inhibit viral release in CCB cells.

Earlier PCR protocols for detection (Gilad et al., 2002, Bercovier et al., 2005) or quantitation (Gilad et al., 2004, Yuasa et al., 2012) of CyHV-3 were developed based on the amplification of regions that either lacked a biologically defined function or on the TK gene. For this study, a new qPCR protocol was developed to measure the release of viral particles from CCB infected cells for a gene (ORF81) with a biologically defined function which was not targeted by the siRNA. Previous findings in TK deletions reported no significant differences for *in vitro* replication of CyHV-3 compared to wild-type virus (Costes et al., 2008, Fuchs et al., 2011). However, differences in mortality rates were observed in the CyHV-3 TK deletion strain during an *in vivo* trial (Costes et al., 2008). The disparity from those trials can lead one to speculate that although attenuation of CyHV-3 in the TK deletion strain can be observed *in vivo* by lower mortality rates, it may have been overlooked *in vitro* because both aforementioned studies measured virus titer by endpoint titration. In this study, viral replication was measured by qPCR, which is a sensitive quantitation tool that recognizes fine difference in copy number. In our study we were able to observe differences of viral particle release by siRNA inhibition of both TK and DP as compared to control siRNA in CyHV-3 infected CCB cells. The subtle differences detected by qPCR may explain differential *in vivo* survival rates observed in TK deletions (Costes et al., 2008). The siRNAs used in this trial were unmodified and are known to be effective for a limited time after transfection (Schyth et al., 2012). The transient nature of siRNA inhibition can be enhanced by chemical modifications to siRNAs (Schyth et al., 2012) or the use of longer dsRNA (27/25 mer) that are cellular substrate targets for dicer (Bohle et al., 2011).

For this study, siRNA was designed to target two genes involved in DNA replication (TK and DP) and the inhibition of viral replication caused by the siRNAs was measured by a reporter gene (ORF81) all of which are considered early genes that are transcribed in the first 2–4 h after CyHV-3 infection in CCB cells (Ilouze et al., 2012). Although the statistical significance was not explicitly calculated for the effectiveness of each siRNA, experiments performed in triplicate showed that treatment with siRNA targeting either TK or DP genes significantly reduced the release of viral particles from infected CCB cells (Fig. 2). However, siRNA targeting DP was most effective at reducing viral release as measured by qPCR of ORF81. This preliminary trial with siRNA establishes the basis for using RNAi technology, preferably more stable forms such as long dsRNA that target the entire DP transcript, to control CyHV-3. Further *in vivo* trials should be performed to determine whether RNAi-based therapeutics can be used to protect fish from CyHV-3.

## Conflict of interest

None of the authors of this paper has a financial and personal relationship with other people and organizations that could inappropriately influence or bias the contents of the paper.

## References

[bib0005] Adamek M., Rakus K.L., Chyb J., Brogden G., Huebner A., Irnazarow I., Steinhagen D. (2012). Interferon type I responses to virus infections in carp cells: in vitro studies on Cyprinid herpesvirus 3 and *Rhabdovirus carpio* infections. Fish Shellfish Immunol..

[bib0010] Adamek M., Syakuri H., Harris S., Rakus K.L., Brogden G., Matras M., Irnazarow I., Steinhagen D. (2013). Cyprinid herpesvirus 3 infection disrupts the skin barrier of common carp (*Cyprinus carpio* L.). Vet. Microbiol..

[bib0015] Aoki T., Hirono I., Kurokawa K., Fukuda H., Nahary R., Eldar A., Davison A.J., Waltzek T.B., Bercovier H., Hedrick R.P. (2007). Genome sequences of three koi herpesvirus isolates representing the expanding distribution of an emerging disease threatening koi and common carp worldwide. J. Virol..

[bib0020] Bercovier H., Fishman Y., Nahary R., Sinai S., Zlotkin A., Eyngor M., Gilad O., Eldar A., Hedrick R.P. (2005). Cloning of the koi herpesvirus (KHV) gene encoding thymidine kinase and its use for a highly sensitive PCR based diagnosis. BMC Microbiol..

[bib0025] Bergmann S.M., Kempter J. (2011). Detection of koi herpesvirus (KHV) after re-activation in persistently infected common carp (*Cyprinus carpio* L.) using non-lethal sampling methods. Bull. Eur. Ass. Fish Pathol..

[bib0030] Bohle H., Lorenzen N., Schyth B.D. (2011). Species specific inhibition of viral replication using dicer substrate siRNAs (DsiRNAs) targeting the viral nucleoprotein of the fish pathogenic rhabdovirus viral hemorrhagic septicemia virus (VHSV). Antiviral Res..

[bib0035] Bretzinger A., Fischer-Scherl T., Oumouna M., Hoffmann R., Truyen U. (1999). Mass mortality in koi carp *Cyprinus carpio*, associated with gill and skin disease. Bull. Eur. Ass. Fish Pathol..

[bib0040] Costes B., Fournier G., Michel B., Delforge C., Raj V.S., Dewals B., Gillet L., Drion P., Body A., Schynts F., Lieffrig F., Vanderplasschen A.F.C. (2008). Cloning of the koi herpesvirus genome as an infectious bacterial artificial chromosome demonstrates that disruption of the thymidine kinase locus induces partial attenuation in *Cyprinus carpio koi*. J. Virol..

[bib0045] Dishon A., Davidovich M., Ilouze M., Kotler M. (2007). Persistence of cyprinid herpesvirus 3 in infected cultured carp cells. J. Virol..

[bib0050] Dishon A., Perelberg A., Bishara-Shieban J., Ilouze M., Davidovich M., Werker S., Kotler M. (2005). Detection of carp interstitial nephritis and gill necrosis virus in fish droppings. Appl. Environ. Microbiol..

[bib0055] Eide K.E., Miller-Morgan T., Heidel J.R., Kent M.L., Bildfella R.J., LaPatra S., Watson G., Jin L. (2011). Investigation of koi herpesvirus latency in koi. J. Virol..

[bib0060] Fire A., Xu S., Montgomery M.K., Kostas S.A., Driver S.E., Mello C.C. (1998). Potent and specific genetic interference by double-stranded RNA in *Caenorhabditis elegans*. Nature.

[bib0065] Fuchs W., Fichtner D., Bergmann S.M., Mettenleiter T.C. (2011). Generation and characterization of koi herpesvirus recombinants lacking viral enzymes of nucleotide metabolism. Arch. Virol..

[bib0070] Gavrilov K., Saltzman M. (2012). Therapeutic siRNA: principles, challenges, and strategies. Yale J. Biol. Med..

[bib0075] Gilad O., Yun S., Andree K.B., Adkison M.A., Zlotkin A., Bercovier H., Eldar A., Hedrick R.P. (2002). Initial characteristics of koi herpesvirus and development of a polymerase chain reaction assay to detect the virus in koi *Cyprinus carpio* koi. Dis. Aquat. Organ..

[bib0080] Gilad O., Yun S., Zagmutt-Vergara F.J., Leutenegger C.M., Bercovier H.V., Hedrick R.P. (2004). Concentrations of a koi herpesvirus (KHV) tissues of experimentally infected *Cyprinus carpio* koi as assessed by real-time TaqMan PCR. Dis. Aquat. Org..

[bib0085] Gotesman M., Abd-Elfattah A., Kattlun J., Soliman H., El-Matbouli M. (2013). Investigating the interactions of Cyprinid herpesvirus-3 with host proteins in goldfish *Carassius auratus*. J. Fish Dis..

[bib0090] Gotesman M., Soliman H., El-Matbouli M. (2013). Antibody screening identifies 78 putative host proteins involved in Cyprinid herpesvirus 3 infection or propagation in common carp, *Cyprinus carpio* L.. J. Fish Dis..

[bib0095] Han J.E., Kim J.H., Renault T., Shin S.P., Jun J.W., Park S.C. (2013). Identifying the viral genes encoding envelope glycoproteins for differentiation of cyprinid herpesvirus 3 isolates. Viruses.

[bib0100] Hedrick R.P., Gilad O., Yun S., Spangenberg J.V., Marty G.D., Nordhausen R.W., Kebus M.J., Bercovier H., Eldar A. (2000). A herpesvirus associated with mass mortality of juvenile and adult koi, a strain of common carp. J. Aquat. Anim. Health.

[bib0105] Ilouze M., Dishon A., Kahan T., Kotler M. (2006). Cyprinid herpes virus-3 (CyHV-3) bears genes of genetically distant large DNA viruses. FFBS Lett..

[bib0110] Ilouze M., Dishon A., Kotler M. (2012). Coordinated and sequential transcription of the cyprinid herpesvirus-3 annotated genes. Virus Res..

[bib0115] Kielpinski M., Kempter J., Panicz R., Sadowski J., Schütze H., Ohlemeyer S., Bergmann S.M. (2010). Detection of KHV in freshwater mussels and crustaceans from ponds with KHV history in common carp (*Cyprinus carpio*). Isr. J. Aquacul..

[bib0120] Kim M.S., Jee B.Y., Cho M.Y., Kim J.W., Jeong H.D., Kim K.H. (2012). Fugu double U6 promoter-driven long double-stranded RNA inhibits proliferation of viral hemorrhagic septicemia virus (VHSV) in fish cell lines. Arch. Virol..

[bib0125] Kim M.S., Kim K.H. (2011). Inhibition of viral hemorrhagic septicemia virus replication using a short hairpin RNA targeting the G gene. Arch. Virol..

[bib0130] Kurita J., Yuasa K., Ito T., Sano M., Hedrick R.P., Engelsma M.Y., Haenen O.L.M., Sunarto A., Kholidin E.B., Chou H-Y., Tung M-C., Pena L.D.L., Lio-Po G., Tu C., Way K., Lida T. (2009). Molecular epidemiology of koi herpesvirus. Fish Pathol..

[bib0135] Lima P.C., Harris J.O., Cook M. (2013). Exploring RNAi as a therapeutic strategy for controlling disease in aquaculture. Fish Shellfish Immunol..

[bib0140] Michel B., Leroy B., Raj V.S., Lieffrig F., Mast J., Wattiez R., Vanderplasschen A.F., Costes B. (2010). The genome of cyprinid herpesvirus 3 encodes 40 proteins incorporated in mature virions. J. Gen. Virol..

[bib0145] Minamoto T., Honjo M.N., Yamanaka H., Tanaka N., Itayama T., Kawabata Z. (2011). Detection of cyprinid herpesvirus-3 DNA in lake plankton. Res. Vet. Sci..

[bib0150] Obbard D.J., Gordon K.H., Buck A.H., Jiggins F.M. (2009). The evolution of RNAi as a defence against viruses and transposable elements. Philos. Trans. R. Soc. B Biol. Sci..

[bib0155] Ouyang P., Rakus K., Boutier M., Reschner A., Leroy B., Ronsmans M., Fournier G., Scohy S., Costes B., Wattiez R., Vanderplasschen A. (2013). The IL-10 homologue encoded by cyprinid herpesvirus 3 is essential neither for viral replication in vitro nor for virulence in vivo. Vet. Res..

[bib0160] Perelberg A., Ronena A., Hutorana M., Smithc Y., Kotler M. (2005). Protection of cultured *Cyprinus carpio* against a lethal viral disease by an attenuated virus vaccine. Vaccine.

[bib0165] Rakus K., Irnazarow I., Adamek M., Palmeira L., Kawana Y., Hirono I., Kondo H., Matras M., Steinhagen D., Flasz B., Brogden G., Vanderplasschen A., Aoki T. (2012). Gene expression analysis of common carp (*Cyprinus carpio* L.) lines during Cyprinid herpesvirus 3 infection yields insights into differential immune responses. Dev. Comp. Immunol..

[bib0170] Ronen A., Perelberg A., Abramowitz J., Hutoran M., Tinman S., Bejerano Y., Steinitz M., Kotler M. (2003). Efficient vaccine against the virus causing a lethal disease in cultured *Cyprinus carpio*. Vaccine.

[bib0175] Rosenkranz D., Klupp B.G., Teifke J.P., Granzow H., Fichtner D., Mettenleiter T.C., Fuchs W. (2008). Identification of envelope protein pORF81 of koi herpesvirus. J. Gen. Virol..

[bib0180] Ruiz S., Schyth B.D., Encinas P., Tafalla C., Estepa A., Lorenzen N., Coll J.M. (2009). New tools to study RNA interference to fish viruses: fish cell lines permanently expressing siRNAs targeting the viral polymerase of viral hemorrhagic septicemia virus. Antiviral Res..

[bib0185] Sano M., Ito T., Kurita J., Yanai T., Watanabe N., Miwa S., Lida T. (2004). First detection of koi herpesvirus in cultured common carp *Cyprinus carpio* in Japan. Fish Pathol..

[bib0190] Sarathi M., Simon M.C., Ahmed V.P., Kumar S.R., Sahul Hameed A.S. (2008). Silencing VP28 gene of white spot syndrome virus of shrimp by bacterially expressed dsRNA. Mar. Biotechnol..

[bib0195] Sarathi M., Simon M.C., Venkatesan C., Thomas J., Ravi M., Madan N., Thiyagarajan S., Sahul Hameed A.S. (2010). Efficacy of bacterially expressed dsRNA specific to different structural genes of white spot syndrome virus (WSSV) in protection of shrimp from WSSV infection. J. Fish Dis..

[bib0200] Schyth B.D., Bramsen J.B., Pakula M.M., Larashati S., Kjems J., Wengel J., Lorenzen N. (2012). In vivo screening of modified siRNAs for non-specific antiviral effect in a small fish model: number and localization in the strands are important. Nucleic Acids Res..

[bib0205] Schyth B.D., Lorenzen N., Pedersen F.S. (2007). A high throughput in vivo model for testing delivery and antiviral effects of siRNAs in vertebrates. Mol. Ther..

[bib0210] St-Hilaire S., Beevers N., Way K., Le Deuff R.M., Martin P., Joiner C. (2005). Reactivation of koi herpesvirus infections in common carp *Cyprinus carpio*. Dis. Aquat. Org..

[bib0215] Uchii K., Telschow A., Minamoto T., Yamanaka H., Honjo M.N., Matsui K., Kawabata Z. (2011). Transmission dynamics of an emerging infectious disease in wildlife through host reproductive cycles. ISME J..

[bib0220] Yuasa K., Kurita J., Kawana M., Kiryu I., Oseko N., Sano M. (2012). Development of mRNA-specific RT-PCR for the detection of koi herpesvirus (KHV) replication stage. Dis. Aquat. Org..

